# An Effective Machine Learning Approach for Identifying Non-Severe and Severe Coronavirus Disease 2019 Patients in a Rural Chinese Population: The Wenzhou Retrospective Study

**DOI:** 10.1109/ACCESS.2021.3067311

**Published:** 2021-03-19

**Authors:** Peiliang Wu, Hua Ye, Xueding Cai, Chengye Li, Shimin Li, Mengxiang Chen, Mingjing Wang, Ali Asghar Heidari, Mayun Chen, Jifa Li, Huiling Chen, Xiaoying Huang, Liangxing Wang

**Affiliations:** 1 Department of Pulmonary and Critical Care MedicineThe First Affiliated Hospital of Wenzhou Medical University Wenzhou 325000 China; 2 Department of Pulmonary and Critical Care MedicineAffiliated Yueqing Hospital, Wenzhou Medical University 26453 Yueqing 325600 China; 3 College of Computer Science and Artificial IntelligenceWenzhou University 26495 Wenzhou 325035 China; 4 Department of Information TechnologyWenzhou Vocational College of Science and Technology 499559 Wenzhou 325006 China; 5 School of Surveying and Geospatial Engineering, College of EngineeringUniversity of Tehran 48425 Tehran 1417466191 Iran; 6 Department of Computer ScienceSchool of ComputingNational University of Singapore 37580 Singapore 117417

**Keywords:** COVID-19, coronavirus, support vector machine, slime mould algorithm, disease diagnosis, feature selection

## Abstract

This paper has proposed an effective intelligent prediction model that can well discriminate and specify the severity of Coronavirus Disease 2019 (COVID-19) infection in clinical diagnosis and provide a criterion for clinicians to weigh scientific and rational medical decision-making. With indicators as the age and gender of the patients and 26 blood routine indexes, a severity prediction framework for COVID-19 is proposed based on machine learning techniques. The framework consists mainly of a random forest and a support vector machine (SVM) model optimized by a slime mould algorithm (SMA). When the random forest was used to identify the key factors, SMA was employed to train an optimal SVM model. Based on the COVID-19 data, comparative experiments were conducted between RF-SMA-SVM and several well-known machine learning algorithms performed. The results indicate that the proposed RF-SMA-SVM not only achieves better classification performance and higher stability on four metrics, but also screens out the main factors that distinguish severe COVID-19 patients from non-severe ones. Therefore, there is a conclusion that the RF-SMA-SVM model can provide an effective auxiliary diagnosis scheme for the clinical diagnosis of COVID-19 infection.

## I. Introduction

On December 31, 2019, the Health Commission of Hubei Province of China notified the World Health Organization (WHO) of a rapidly evolving outbreak of unexplained viral pneumonia, which is now believed to have begun since December 1, 2019 [1]. On February 11, 2020, this viral pneumonia has been named ‘coronavirus disease 2019’ (COVID-19) by the WHO [2]. At the same time, according to the recommendations from the International Committee on Taxonomy of Viruses (ICTV), the 2019 novel coronavirus was named as ‘severe acute respiratory syndrome coronavirus 2’ (SARS-CoV-2) [3]. By Mar 23th, 2020, a total of 332,930 laboratory-confirmed cases of SARS-CoV-2 infection have been reported, including 14,510 deaths [4]. Therefore, COVID-19 has been described as a global pandemic [5], [6].

COVID-19 is characterized by a combination of clinical features that include fever, dry cough, sputum, myalgia, tachypnea, fatigue, lymphopenia and abnormal chest computed tomography (CT) imaging [1], [7], [8]. With the improvement of accuracy on the diagnosis of patients with COVID-19, prediction of outcome in critically ill patients with COVID-19 is becoming a more and more important and urgent issue for treatment decisions and resource allocation at high-risk infection regions. A growing number of clinical studies have shown 26.1-32% of patients with COVID-19 develop severe COVID-19, and severe COVID-19 is characterized by rapid progression, severe acute respiratory disease syndrome (ARDS), multiple organs dysfunction, and high mortality [1], [7]. The previous study has described that elderly patients are more susceptible to COVID-19 infection, especially those with diabetes, hypertension, chronic pulmonary diseases, and cerebral infarction [9]. Likewise, a high fatality rate (61.5%) was observed among 52 critically ill adult patients with COVID-19 in Wuhan, China [10]. Due to COVID-19 rapid progression, a demand for intensive care unit beds is rising in a short period of time when the resource is constrained. Hence, compared to rescue and treatment for critically ill patients, early identification and intervention play a more indispensable role in preventing COVID-19 from the mild to severe. If effective novel prognostic model systems are established in time, would help to surveil the status of patients with COVID-19, with the potential for mortality reduction.

So far, it is recognized that COVID-19 is a disease involving multiple situations. Its clinical manifestation is complicated and changeable, and the critically ill cases rapidly develop, especially in patients with acute respiratory disease syndrome [11]. However, the survival rate of critically ill cases remains low, with the rising number of critically ill patients, and the focus on controlling of the infection source and cutting off the route of transmission in health and epidemic prevention work has shifted to timely detection and effective assessment of critically ill patients, and early intervention before fatal events appear. Currently, the diagnosis of critically ill and fatal cases is usually based on a large number of clinical laboratory parameters and clinical characteristics through judgment calls. Therefore, misdiagnosis and missed diagnosis easily exist because of physicians’ fatigue, pressure, lack of experience, inconsistent judgments and lack of prior knowledge. Continuously, COVID-19 outbreaks are operating, worldwide, and meanwhile the rates of misdiagnosis and missed diagnosis are becoming the world’s most closely watched numbers.

In the present study, the data was retrospectively collected from patients with COVID-19 pneumonia who were admitted to the Affiliated Yueqing Hospital of Wenzhou Medical University from Jan 21 to Mar 10, 2020, in a single-center retrospective study. Severe acute respiratory syndrome coronavirus 2 (SARS-CoV-2) was detected by real-time reverse transcriptase-polymerase chain reaction (RT-PCR). RT-PCR assay kit was provided by Huada Genomics Co., Ltd. Throat swabs from all 51 patients tested positive for SARS-CoV-2 by RT-PCR. According to the “the New Coronavirus Pneumonia Prevention and Control” promulgated by the National Health Commission of the People’s Republic of China, in 2020, all patients fulfilled diagnostic criteria of COVID-19 pneumonia. To simplify the analysis, the 51 COVID-19 patients were divided the 51 COVID-19 patients into two groups: severe COVID-19 (n = 21) and non-severe COVID-19 (n = 30). Severe COVID-19 diagnosis should meet at least 1 of the following criteria: (1) Respiratory distress and the respiratory rate over 30 breaths/min; (2) Resting oxygen saturation lower than 93%; (3) Oxygenation index (OI) below 300 mmHg. The research plan was permitted by the Ethics Committees of the Affiliated Yueqing Hospital of Wenzhou Medical University (Yueqing, China) (Ethical approval code: 202000002). This study was in compliance with the Helsinki declaration. Blood samples were collected from all COVID-19 patients and blood routine examination was conducted, using the whole blood by a Mindray BC-5300 automatic blood cell analyzer (Mindray, Shenzhen, China). In this study, the basic information of the COVID-19 patients and 23 blood routine parameters (features) are listed in Table 1.

**TABLE 1:** List of the Features Used in This Study and Their Definitions

Number	Features	Abbreviation
F1	Gender	Gender
F2	Age	Age
F3	White blood cell	WBC
F4	Neutrophil percentage	NEU%
F5	Lymphocyte percentage	LY%
F6	Monocyte percentage	MONO%
F7	Eosinophils percentage	EOS%
F8	Basophils percentage	BA%
F9	Neutrophil count	NEU
F10	Lymphocyte count	LY
F11	Monocyte count	MONO
F12	Eosinophils count	EOS
F13	Basophils count	BA
F14	Red blood cell	RBC
F15	Haemoglobin	HB
F16	Hematocrit	HCT
F17	Mean corpuscular hemoglobin	MCH
F18	Mean corpuscular volume	MCV
F19	Mean corpuscular hemoglobin concentration	MCHC
F20	Red blood cell distribution width	RDW
F21	Red blood cell distribution width-SD	RDW-SD
F22	Mean platelet volume	MPV
F23	Blood platelet	PLT
F24	Plateletcrit	PCT
F25	Platelet distribution width	PDW
F26	Platelet-large cell ratio	P-LCR%,
F27	Neutrophil to Lymphocyte ratios	N/L
F28	Platelet to Lymphocyte ratios	P/L

In recent years, the development and application of machine learning in term of infectious disease have been beneficial for the prevention and control of infectious diseases. Machine learning methods play more and more significant roles in in a wide fields of medical science [12], [13], which is widely performed in disease diagnosis, development of prediction models and identification of significant risk factors [14]. Undoubtedly, machine learning methods have some advantages including improving health professionals’ diagnostic capacities, reducing much of the workload of radiologists and anatomic/clinical pathologists, enhancing the diagnostic accuracy [15]. Therefore, machine learning has emerged as an indispensable tool for physicians to lead a better understanding for patient personalized treatments. In the meantime, it is hoped that machine learning methods based on the abundant clinical data will support researches into COVID-19 epidemiology, which can cope with the current disease outbreaks [16]. Therefore, machine learning based on clinical characteristics is a promising method for the prediction of survival rate and classification of disease severity. To this end, a new method with the random forest (RF) and slime mould algorithm (SMA) optimized support vector machines (SVM) model involved was established, to analyze data of 51 patients confirmed with COVID-19 pneumonia who were admitted to the Affiliated Yueqing Hospital of Wenzhou Medical University (Yueqing, China).

Since then, several machine learning methods have been taken to tackle the related problems of COVID-19. For example, Wang *et al.* [17] firstly used a Respiratory Simulation Model (RSM) to fill the gap between large amounts of training data and scarce real-world data. A GRU neural network with bidirectional and attentional mechanisms (BI-AT-GRU) was then operated to classify 6 key clinically respiratory patterns for screening large-scale individuals infected with COVID-19. Hu *et al.* [18] developed an improved stacked autoencoder for modeling epidemic transmission dynamics and applying this model to predicting COVID-19 confirmed cases in real-time across China. Butt *et al.* developed a three-dimensional deep learning model by using lung CT image sets to segment candidate infection areas, and then utilizing a location attention classification model to classify these separated images into COVID-19, influenza A viral pneumonia, and healthy cases. Wu *et al.* [19] performed a calibration of logistic growth model, and generalized logistic growth model, growth model, and Richards model on the number of reported infection cases in the severely affected areas. Different models provide predictions with different upper and lower limits. Shan *et al.* [20] developed a deep learning-based segmentation system to automatically quantify possible infected areas and their volume ratios in the lungs. At the same time, a human-in-the-loop (HITL) strategy was used to assist the radiologist in segmenting the infected area, thereby shortening the total segmentation time to 4 minutes. Yang *et al.* [21] integrated population migration data and the latest COVID-19 epidemiological data into the Susceptible-Exposed-Infectious-Removed (SEIR) model to obtain the epidemic curve, and also used artificial intelligence (AI) derived from 2003 SARS data ways to predict the next outbreak. Vivanco-Lira [22] tried to summarize the biological characteristics of COVID-19 virus and the possible pathophysiology of its disease, as well as a random model that characterizes the probability distribution of cases in Mexico and the number of Mexican cases estimated by differential equation models. Gozes *et al.* [23] proposed a method that leverages powerful 2D and 3D deep learning models to modify and adjust existing AI models, and combined them with clinically understood systems, and used 3D volumetric examinations to assess each patient’s disease progression over time.

In this study, it aims to propose efficient models by using the SMA, which is employed to train an effective SVM model. Then, it is the first time that the optimized SMA-SVM is employed to diagnose the severity of COVID-19. The active model is built by using the info about patients’ basic information and 26 blood routine indicators. In the developed model (SMA-SVM), SMA was used for training an SVM model and random forest was employed to identify the most informative risk factors of COVID-19 infected people at the same time. In the experiment, machine learning methods such as random forest (RF) combined with SMA-SVM (RF-SMA-SVM), RF combined with Harris hawks optimization-based SVM (RF-HHO-SVM), RF combined with moth-flame optimization-based SVM (RF-MFO-SVM), RF combined with particle swarm optimization-based SVM (RF-PSO-SVM), RF combined with grid search-based SVM (RF-Grid-SVM) and SMA-SVM were rigorously compared. The results showed that the established RF-SMA-SVM method performed far better than its peers on four evaluation metrics: classification accuracy (ACC), sensitivity, specificity and Matthews correlation coefficient (MCC). Therefore, the main contributions of this study can be listed below.

a) Successful applications of SMA are for parameter tuning of SVM.
b) The proposed RF-SMA-SVM was the first time to apply in the diagnosis of the severity of COVID-19.
(c) It is the first time that the age, Neutrophil-to-Lymphocyte ratio (N/L) and Platelet-to-lymphocyte ratio (P/L) combined together for predicting the prognosis of patients with COVID-19.
d) The established RF-SMA-SVM model outperforms other competitors.

The structure of the paper is as bellow. Section 2 describes the data and proposed the RF-SMA-SVM model in detail. The experimental setup and results are presented in Section 3. Section 4 presents the discussions. The conclusions and future works are given in Section 5.

## II. Materials and Methods

### A. Data Collection

**FIGURE 1.**
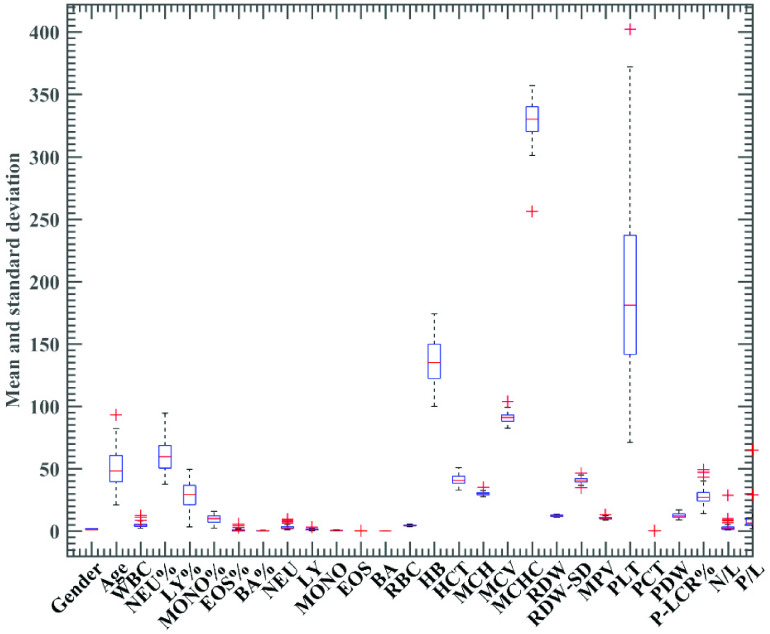
Boxplot of the 28 features.

The boxplot of total 28 indexes are shown in Figure 1. Statistical analyses were performed by using SPSS version 21.0 (IBM, Somers, NY, US). Continuous variables (age and blood routine parameters) were analyzed by an independent sample t-test. Results were considered statistically significant with p < 0.05. Detailed results of the statistical analysis are shown in Table 2.

**TABLE 2:** Statistical Analysis of 51 COVID-19 Patients (Severe COVID-19 and Non-Severe COVID-19)

Index	Severe (n = 21)	Non-severe (n = 30).	p-value
Age (years)	61.43±17.64	42.30±11.53	0.00
WBC (10^9/L)	5.06±2.23	4.79±1.89	0.64
NEU (%)	69.50±14.10	55.33±9.98	0.00
LY (%)	21.20±10.54	33.25±7.90	0.00
MONO (%)	8.71±4.04	10.35±3.19	0.11
EOS (%)	0.44±1.22	0.88±0.96	0.16
BA (%)	0.15±0.19	0.19±0.13	0.38
NEU (10^9/L)	3.68±2.22	2.74±1.59	0.08
LY (10^9/L)	0.95±0.46	1.50±0.45	0.00
MONO (10^9/L)	0.40±0.19	0.48±0.18	0.15
EOS (10^9/L)	0.01±0.03	0.04±0.04	0.02
BA (10^9/L)	0.01±0.01	0.01±0.01	0.22
RBC (10^12/L)	4.44±0.66	4.58±0.47	0.35
HB (g/L)	132.67±18.68	138.53±14.71	0.22
HCT (%)	40.25±4.92	41.83±3.87	0.21
MCH (pg)	29.652±1.22	30.28±1.47	0.11
MCV (fL)	90.35±3.97	91.54±4.08	0.30
MCHC (g/L)	323.76±21.23	330.87±10.77	0.12
RDW (%)	12.48±0.55	12.083±0.58	0.02
RDW-SD (fL)	41.27±1.74	40.19±2.47	0.09
MPV (fL)	10.42±0.92	10.43±0.95	0.97
PLT (10^9/L)	170.14±63.10	205.30±69.82	0.07
PCT (%)	0.19±0.07	0.21±0.07	0.26
PDW (%)	12.62±2.29	12.77±2.01	0.81
P-LCR (%)	28.14±7.35	28.10±7.09	0.98
N/L	5.35±5.97	1.85±0.86	0.02
P/L	12.53±13.63	6.52±2.62	0.02

### B. RF-SMA-SVM Method

The main flow of the proposed method is illustrated in Figure 5. It consists of four steps: data acquisition with standardization, feature selection, model training, and classification. The first step is to normalize the acquired data to the range of [−1, 1], and then use the random forest algorithm to select the feature set with the highest score from the data set and SMA for training the parameters ($C, \gamma$) in the SVM. The third step uses the optimized parameters and the feature set selected by the random forest to train the SVM again. The last step is to use the trained SVM for classification of new data. When evaluating the trained model, K-fold cross-validation (CV) is used to obtain less biased experimental results. In this experiment, K is set to 10.

**FIGURE 2.**
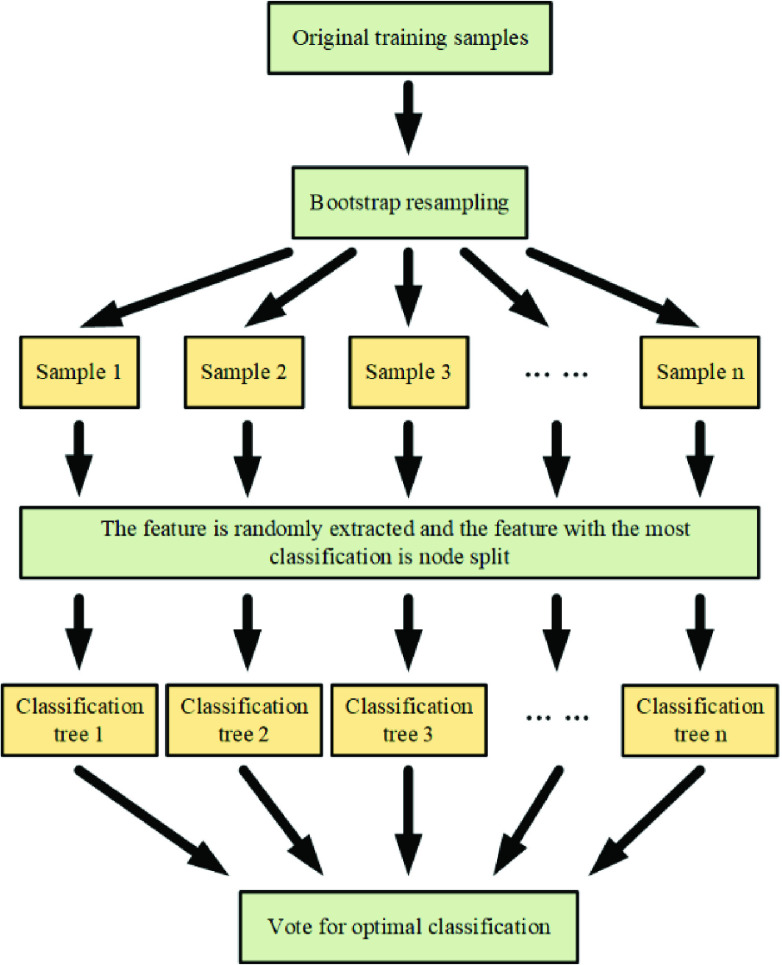
Flowchart of RF.

**FIGURE 3.**
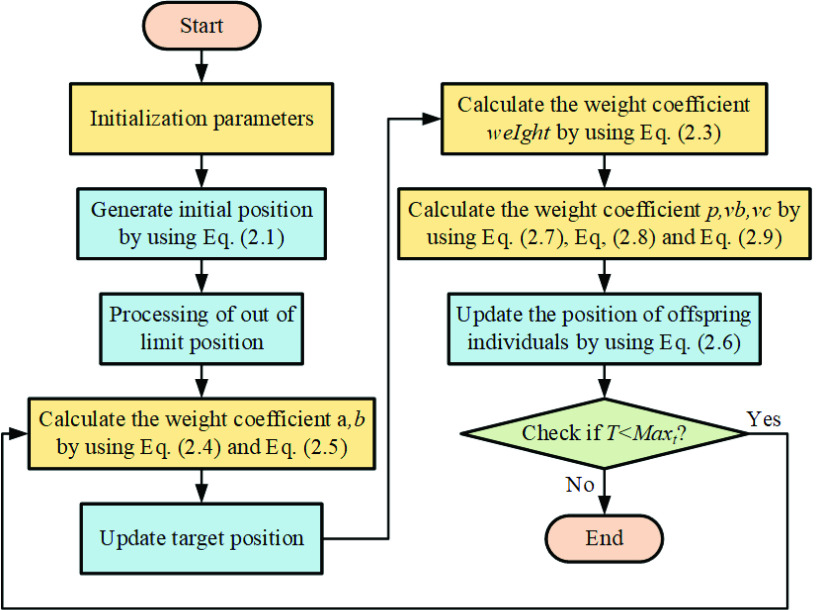
Flowchart of SMA.

**FIGURE 4.**
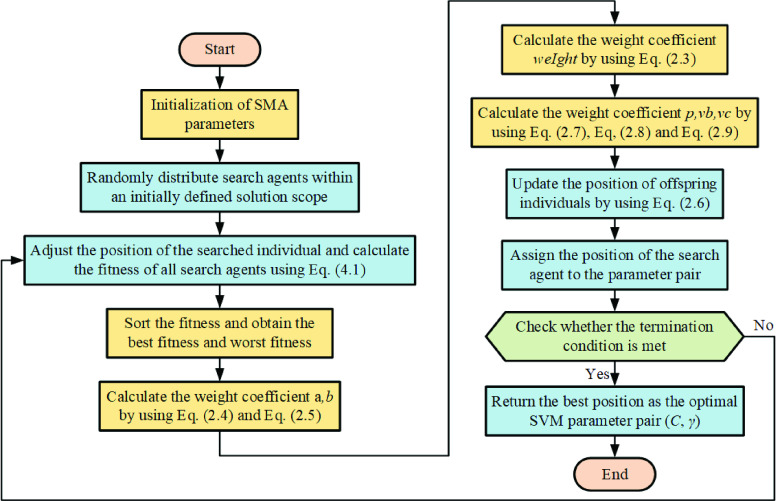
Flowchart of SMA-SVM.

**FIGURE 5.**
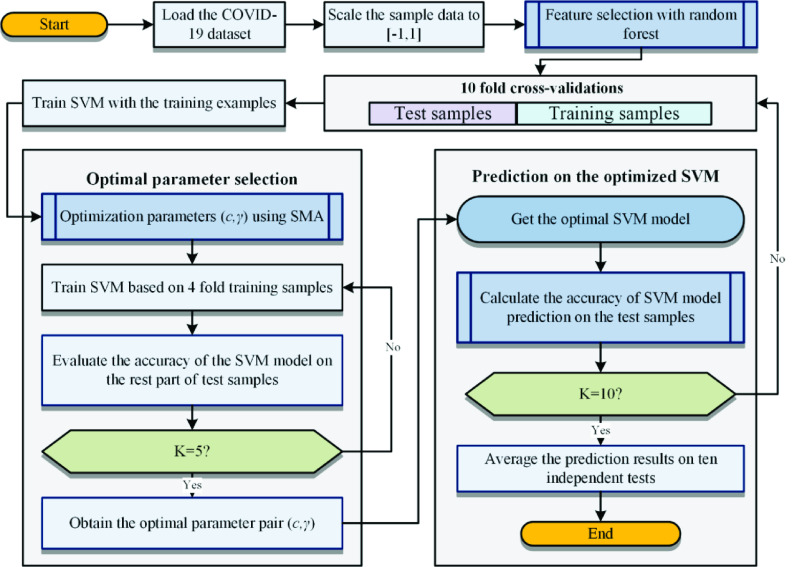
Flowchart of RF-SMA-SVM.

#### 1) Feature Selection Via Random Forest

Random forest (RF) [24], is a combined classification model consisting of multiple decision tree models. It employs bootstrap resampling to take multiple samples from the original sample and models a decision tree for each bootstrap sample. Then, these decision trees are combined together, and the final classification or prediction result is obtained by voting. In addition, a large number of theoretical and empirical studies have shown that RF not only has high prediction accuracy and excellent tolerance to outliers and noise, but also has the ability to calculate the importance of a single feature and avoid easily overfit.

The steps of calculating the importance of a feature by using RF is shown in Figure 2.

#### 2) Parameter Optimization by SMA

Swarm intelligent algorithms can always generate better solutions than the traditional gradient descent-based methods [25]–[27], SMA as a new member of the swarm intelligent algorithms, it has shown great effectiveness in many fields. SMA mainly simulates the behaviour mode of Physarum polycephalum in slime mould [28]. SMA mainly uses the weighting factor to simulate the changes of the vein structure and contraction pattern of slime mould during foraging. When the concentration of nearby food is large, the diameter of slime bacteria will increase accordingly. When the concentration of food decreases, the diameter of slime bacteria will also shrink, forming a tendency to approach food. The steps can be described by the following steps:

Step 1: Initialization parameters and individual positions. Such as population number $N$, dimension $D$, parameter $z$ and randomly distribute the search agents in the solution space.
  \begin{equation*} X\left ({i,: }\right)=\text {rand}\cdot \left ({\text {UB}-\text {LB} }\right)+\text {LB}\tag{2.1}\end{equation*}
  where $X_{i}$ represents the $i-th$ search agent, rand represents a random number with a value between 0 and 1, and LB and UB represent the lower and upper boundaries of the search space, respectively.
Step 2: Calculate fitness value and weight. First calculate the fitness value of each search agent, sort all fitness values $F$ to get $S$ and $I$, and get the best fitness values bF and wF from them. Then calculate the weight $W$.
  \begin{align*} \left [{ S,I }\right]=&\text {sort}(F)\tag{2.2}\\ { \mathord {\stackrel {{\lower 3pt\hbox {$\displaystyle \rightharpoonup $}}} W}}(I(i),j)=&\begin{cases} 1+r\cdot \log \left ({\frac {\text {bF}-S\left ({i }\right)}{\text {bF}-\text {wF}}+1 }\right),i\le \frac {N}{2}\\ 1-r\cdot \log \left ({\frac {\text {bF}-S\left ({i }\right)}{\text {bF}-\text {wF}}+1 }\right),i>\frac {N}{2} \\ \end{cases}\tag{2.3}\end{align*}
  where $i=1\ldots N,j=1\ldots D$.
Step 3: Update the global optimal fitness value DF and the best search agent position BP (parameter to be optimized). If the fitness value obtained during the current iteration process is better than the global optimal fitness value, the global optimal fitness value is updated to the currently obtained fitness value and the best search agent position is updated to the currently obtained position.
Step 4: Update search agent location (parameter value to be optimized). First update the following parameters:
  \begin{align*} a=&\text {atanh}\left ({-\frac {T}{{\text {Max}}_{\text {t}}}+1 }\right)\tag{2.4}\\ b=&1-\frac {T}{\text {Max}_{\text {t}}}\tag{2.5}\end{align*}
  where $T$ is the current number of iterations and $\text {Max}_{\text {t}}$ is the maximum number of iterations.

Then update the search agent location according to the following formula:

\begin{align*} \overrightarrow {X^{\ast }}=\begin{cases} \text {rand}\cdot \left ({\text {UB}-\text {LB} }\right)+\text {LB},\text {rand} < z \\ \overrightarrow {\text {BP}}(t)+ \overrightarrow {\text {vb}}\cdot \left ({W\cdot \overrightarrow {X_{A}}\left ({t }\right)- \overrightarrow {X_{B}}\left ({t }\right) }\right),r < p \\ \overrightarrow {\text {vc}}\cdot \overrightarrow {X}\left ({t }\right),r\ge p \end{cases}\tag{2.6}\end{align*}

where A and B are two individuals randomly selected from the group, and the formula for $p$, $\overrightarrow {\text {vb}}$ and $\overrightarrow {\text {vc}}$ are as follows:

\begin{align*} p=&\tanh \left |{ S\left ({i }\right)-\text {DF} }\right |\tag{2.7}\\ \overrightarrow {\text {vb}}=&[-a,a]\tag{2.8}\\ \overrightarrow {\text {vc}}=&[-b,b]\tag{2.9}\end{align*}

Figure 3 shows the flowchart of SMA.

#### 3) Classification Based on SVM

SVM is a popular machine learning method that has shown effectiveness in theory and applications [29]–[36]. SVM uses the principle of non-linear mapping: $\Phi:R^{n}\to H$, maps nonlinear eigendata to high dimensional space, thus using linear methods to solve nonlinear problems. For a load sample data set $D=\left \{{ x_{i},y_{i} }\right \},i\in \,\,1,2,\ldots,n,x_{i}\in R^{n},y_{i}\in R$ where $y_{i}$ is the load value of the output and $x_{i}$ is the eigenvector corresponding to the output value, SVM maps the eigenvector $x_{i}$ to high dimensional space by function $\varphi (x)$. Establish the regression function:

\begin{equation*} y=\omega \cdot \varphi \left ({x }\right)+b\tag{3.1}\end{equation*}

where $\omega $ is the weight, $b$ is the intercept, $x=(x_{1},x_{2},\ldots,x_{n})$ is the eigenvector, $y=(y_{1},y_{2},\ldots,y_{n})$ is the output load value.

To find a suitable set of $\omega $ and $b$, the search process was transformed into an optimization problem according to the principle of structural risk minimization. The objective function and constraints are as follows:

\begin{align*} \begin{cases} \min {\frac {1}{2}\left \|{ \omega }\right \|^{2}+C\sum \limits _{i=1}^{n} {(\xi _{i}+\xi _{i}^{\ast })}} \\ s.t.\begin{cases} \omega \cdot \varphi \left ({x_{i} }\right)-y_{i}+b\le \varepsilon +\xi _{i}^{\ast } \\ y_{i}-\omega \cdot \varphi \left ({x_{i} }\right)-b\le \varepsilon +\xi _{i} \\ \xi _{i}^{\ast }\ge 0,\xi _{i}\ge 0,i=1,2,\ldots,n \\ \end{cases} \end{cases}\tag{3.2}\end{align*}

where $C$ is the penalty factor, $\xi _{i}$ and $\xi _{i}^{\ast }$ are relaxation coefficients, allowing a certain fitting error, $\varepsilon $ is the maximum error, and is a linear insensitive loss function parameter.

For the sake of enhancing the efficiency of optimization, Lagrange multiplier method is applied to transform it into dual problem:

\begin{align*} \begin{cases} \min \frac {1}{2}\sum \limits _{i,j=1}^{n} \left ({a_{i}^{\ast }-a_{i} }\right)\left ({a_{j}^{\ast }-a_{j} }\right)K\left ({x_{i},x_{j} }\right)\\ \qquad \quad +\varepsilon \sum \limits _{i=1}^{n} {(a_{i}^{\ast }+a_{i})} -\sum \limits _{i=1}^{n} {y_{i}(a_{i}^{\ast }-a_{i})} \\ s.t.\begin{cases} \sum \limits _{i=1}^{N} \left ({a_{i}-a_{i}^{\ast } }\right) =0 \\ 0\le a_{i},a_{i}^{\ast }\le C,i=1,2,\ldots,n \\ \end{cases} \end{cases}\tag{3.3}\end{align*}

where $a_{i}$ and $a_{i}^{\ast }$ are Lagrange multipliers, $K\left ({x_{i},x_{j} }\right)$ is a kernel function. Gaussian radial basis kernel function was employed:

\begin{equation*} K\left ({x_{i},x_{j} }\right)=\text {exp}(-{\gamma \left \|{ x_{i}-x_{j} }\right \|}^{2})\tag{3.4}\end{equation*}

where $\gamma $ is a kernel parameter. Eventually, the nonlinear function was obtained.

\begin{equation*} y=\sum \limits _{i=1}^{n} {\left ({a_{i}-a_{i}^{\ast } }\right)K\left ({x_{i},x_{j} }\right)} +b\tag{3.5}\end{equation*}

In SVM, the penalty factor $C$ and the kernel parameter $\gamma $ have a crucial impact on the learning process, and the prediction effects obtained by different parameters are quite diverse. $C$ indicates the generalization ability and error size of SVM. Larger $C$ will make the model error larger and the precision is not enough. Smaller $C$ will lead to weaker generalization ability. $\gamma $ affects the fit of the model. In order to find a better set of $C$, $\gamma $ and improve the classification accuracy of SVM, this paper combined SMA and SVM to produce a new classification model SMA-SVM and applied the model to the prediction problem in reality.

#### 4) Proposed SMA-SVM

To explore the potential of SVM, its parameters are tuned via the SMA. Here, we show the flowchart of parameter optimization of the proposed SMA-SVM model.

In Figure 4, adjust the position of the searched individual and calculate the fitness of all search agents using SVM classifier with the parameter pair according to the following formula:

\begin{equation*} \text {fitness}=\frac {\sum \nolimits _{i=1}^{K} {\text {acc}_{i}}}{K}\tag{4.1}\end{equation*}

## III. Experimental Setup and Results

The entire experiment was built in a MATLAB environment. The main methods involved in the comparison experiments include RF-SMA-SVM and RF-HHO-SVM, RF-MFO-SVM, RF-PSO-SVM, RF-Grid-SVM, SMA-SVM. The optimization methods including HHO, MFO, PSO, SMA, etc. were implemented using the default parameters from the original paper, while the SVM model and RF methods were implemented using the LIBSVM toolbox and RF toolbox respectively.^1^ The input data were normalized to the range [−1, 1] before classification. The maximum number of iterations and the number of group members are set to 50 and 20, respectively. The search range for the two parameters in the SVM (penalty factor $C$ and kernel parameter $\gamma$) is set to $\left [{ 2^{-5},2^{5} }\right]$.^1^https://www.csie.ntu.edu.tw/~cjlin/libsvm/, https://code.google.com/archive/p/randomforest-matlab

**FIGURE 6.**
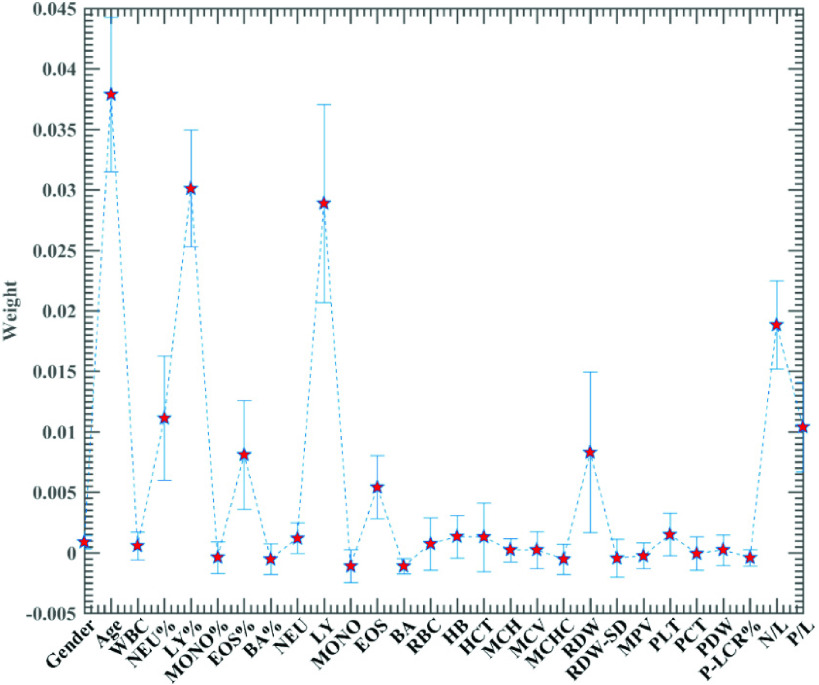
Importance of features obtained by RF.

In this experiment, the RF method was first time to use in assessment of the significant features, and the specific results are shown in Figure 6. As can be seen from the figures, the individual features exhibit different levels of importance, with the top eight most vital features being Age, LY%, LY, N/L, NEU%, P/L, RDW, EOS%, respectively. Sorting this feature sequence in descending order of importance yields with multiple feature combinations, was employed to be evaluated one by one. Considering the number of features, here we select a set of eight incremental features consisting of the first eight features for the experiment. The results of the are shown in Table 3. It can be seen from the table that the best classification accuracy is achieved when the features consisting of the first 6 features.

**TABLE 3:** Classification Performance of RF-SMA-SVM Based on Eight Feature Sets

No.	Feature subset	ACC	MCC	Sensitivity	Specificity
No.1	{Age}	0.783	0.577	0.650	0.867
No.2	{Age, LY%}	0.807	0.640	0.683	0.900
No.3	{Age, LY%, LY}	0.787	0.573	0.617	0.900
No.4	{Age, LY%, LY, N/L}	0.810	0.641	0.733	0.867
No.5	{Age, LY%, LY, N/L, NEU%}	0.843	0.704	0.800	0.867
No.6	{Age, LY%, LY, N/L, NEU%, P/L}	0.900	0.812	0.850	0.942
No.7	{Age, LY%, LY, N/L, NEU%, P/L, RDW}	0.887	0.801	0.833	0.933
No.8	{Age, LY%, LY, N/L, NEU%, P/L, RDW, EOS%}	0.843	0.688	0.750	0.900

The detailed results of RF-SMA-SVM model are shown in Table 4. In order to ensure the accuracy of the results, we repeated 10 times, taking the average of the 10 results as the final result. The results showed that the classification accuracy (ACC) of RF-SMA-SVM was 90.10%, the Matthews correlation coefficient (MCC) was 82.18%, the sensitivity was 88.17%, the specificity was 91.41%, and the variances were 0.1245, 0.2247, 0.2210 and 0.1788, respectively. In addition, it can be observed that the SMA can automatically obtain the optimal parameters of SVM on the optimal feature space obtained from RF, which demonstrates the strong search ability of SMA. the strong search ability of SMA.

**TABLE 4:** Classification Performance of RF-SMA-SVM in Terms of ACC, MCC, Sensitivity, and Specificity

Num	ACC	MCC	Sensitivity	Specificity
No.1	0.9000	0.8116	0.8500	0.9417
No.2	0.9000	0.8220	0.9000	0.9000
No.3	0.9030	0.8150	0.9000	0.9000
No.4	0.9070	0.8280	0.8500	0.9330
No.5	0.9030	0.8320	0.9170	0.9000
No.6	0.9000	0.8220	0.9000	0.9000
No.7	0.9000	0.8250	0.8500	0.9330
No.8	0.9070	0.8280	0.8500	0.9330
No.9	0.9030	0.8320	0.9170	0.9000
No.10	0.8870	0.8130	0.8830	0.9000
AVG.	0.9010	0.8218	0.8817	0.9141
STD	0.1245	0.2247	0.2210	0.1788

**FIGURE 7.**
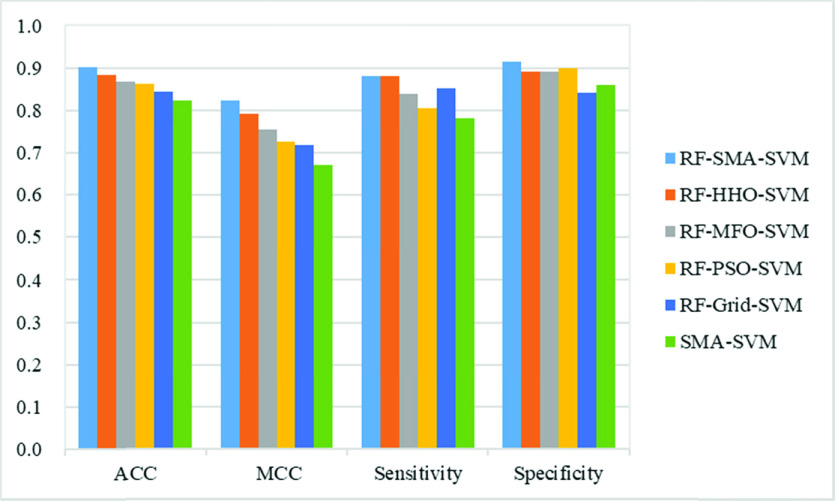
Classification efficacy of six models based on the four metrics.

To verify the validity of this approach, a comparative study presents with five other valid machine learning models, including RF-HHO-SVM, RF-MFO-SVM, RF-PSO-SVM, RF-Grid-SVM, and SMA-SVM. The comparison of six methods is shown in Figure 7. The results showed that the RF-SMA-SVM model outperformed the RF-HHO-SVM, RF-MFO-SVM, RF-PSO-SVM, RF-Grid-SVM, and SMA-SVM models in terms of ACC, MCC, and specificity, with the least variance. Besides, RF-SMA-SVM outperformed RF-MFO-SVM, RF-PSO-SVM and SMA-SVM in sensitivity evaluation metric had lower variance than these three models. This means that the RF-SMA-SVM model gets better classification performance and stability compared to other optimization methods. The RF-SMA-SVM model had the best results on the ACC evaluation metric, 2 percentage points higher than the RF-HHO-SVM, which ranked second. This is followed by RF-MFO-SVM, RF-PSO-SVM, RF-Grid-SVM, and SMA-SVM, with SMA-SVM being 8 percentage points lower than RF-SMA-SVM, and RF-SMA-SVM having the smallest variance of 0.1245, indicating that the RF-SMA-SVM model is more stable in resolving this problem after conduction of feature selection by RF. For the MCC evaluation indicator, the RF-SMA-SVM model still achieved the best results, followed by RF-HHO-SVM, RF-MFO-SVM, RF-PSO-SVM and RF-Grid-SVM, and otherwise SMA-SVM performed the worst, with 15.1 percentage points lower than RF-SMA-SVM. In addition, RF-SMA-SVM had the lowest variance of 0.1992. In terms of sensitivity evaluation indicators, the RF-HHO-SVM and RF-SMA-SVM models were the most effective, followed by the RF-Gird-SVM model with a difference of 3 percentage points. This is followed by RF-MFO-SVM and RF-PSO-SVM, with RF-MFO-SVM being 3.33 percentage points higher compared to RF-PSO-SVM. Moreover, the SMA-SVM model had the worst results and RF-PSO-SVM had the largest variance of 0.2770. In terms of specificity evaluation indicator, the RF-SMA-SVM model has the best results, followed by RF-PSO-SVM, which differs from RF-SMA-SVM by 1.5 percentage points. RF-HHO-SVM, RF-MFO-SVM followed closely, with SMA-SVM being 5.4 percentage points lower than RF-SMA-SVM, RF-Grid-SVM having the largest variance of 0.212 and RF-PSO-SVM having the smallest variance of 0.1688.

**FIGURE 8.**
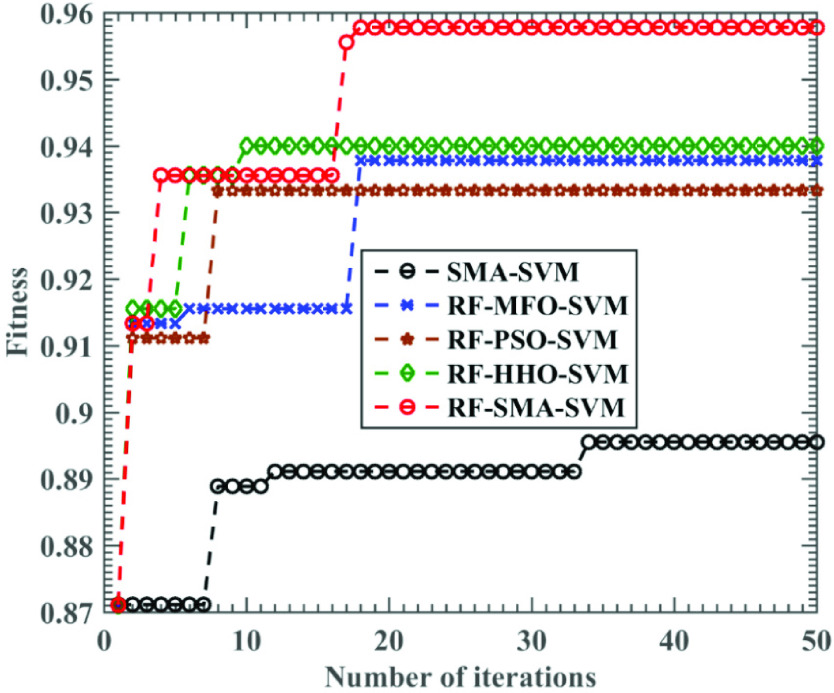
Relationship between training accuracy of several SVM variants and the number of iterations.

The effect of original SMA in test function is far better than PSO and MFO [28], which indicates that SMA has strong exploration and development ability, and these abilities can also play an important role in medical data analysis. Figure 7 and figure 8 prove that RF-SMA-SVM is superior to RF-HHO-SVM, RF-MFO-SVM and RF-PSO-SVM. Although RF-SMA-SVM is slightly inferior to RF-HHO-SVM in sensitivity, it does not affect the superiority of RF-SMA-SVM. Furthermore, 1 is the upper limit to measure these indicators. If you want to be close to 1, the performance requirements of the algorithm are multiplied. Therefore, RF-SMA-SVM seems to have little difference from other comparison algorithms, but in fact there is a certain gap.

To describe the convergence of the proposed RF-SMA-SVM method, we also document the trend of the accuracy of the four SVM models with iteration. In Figure 8, it is found that after several iterations, SMA-SVM has the worst effect, and the accuracy of SMA-SVM does not improve significantly with iterations, so it is easy to fall into the local optimum, while RF-SMA-SVM model can quickly and continuously jump out of the local optimum to reach the optimal accuracy, which shows that RF-SMA-SVM method has strong local search ability and global search ability. The accuracy of RF-MFO-SVM and RF-PSO-SVM is slightly lower than that of RF-SMA-SVM, and the accuracy increases less significantly with iteration, which makes it easier to fall into local optimization.

At the end of the experiment, we compare the computing time of RF-SMA-SVM, RF-MFO-SVM, RF-PSO-SVM and RF-HHO-SVM. The results are shown in Table 5.

**TABLE 5:** Calculation Time Comparison Between RF-SMA-SVM and Other Algorithms

Algorithm	Relative time	Rank
RF-SMA-SVM	4.597	4
RF-MFO-SVM	1.097	2
RF-PSO-SVM	1.000	1
RF-HHO-SVM	1.290	3

The calculation time of RF-PSO-SVM is the shortest, so the relative calculation time of RF-PSO-SVM is recorded as 1. The relative calculation time of other algorithms is calculated according to the calculation time of RF-PSO-SVM. In Table 5, although RF-SMA-SVM has the longest computation time, its accuracy is better than other algorithms.

## IV. Discussions

In the present study, COVID-19 patient datasets were screened by using the random-forest based feature selection method. Importantly, several key features were screened, including age, NEU%, LY%, LY, N/L and P/L. Subsequently, a SMA-SVM model is constructed to accurately assess the severity of the COVID-19 and early monitor COVID-19 progression. Therefore, we believe that RF-SMA-SVM model may help inform clinical decision-making.

Some studies demonstrate that advanced age is a strong predictor for prognosis of Severe Acute Respiratory Syndrome (SARS) and Middle East Respiratory Syndrome (MERS). For instance, Choi and colleagues used multivariable analysis to identify age older as independent predictors of mortality [37]–[39]. In line with these findings from clinical studies, studies on macaques model revealed that older macaques inoculated with SARS coronavirus (SARS-CoV) exhibited stronger host antiviral response compared to younger macaques, which is tightly related to inflammation-related genes and type I interferon [40]. Similarly to these studies, Zhou *et al.* found that non-survivor with COVID-19 had a significantly older median age, than did the survivor with COVID-19 [41]. We also found in our study that patients with severe COVID-19 had significantly higher (1.45-fold) age than those with a non-severe COVID-19 $(\text {P}=0.00)$, suggesting that disease severity may be predicted by age in COVID-19 patients. In addition, numerous clinical studies have demonstrated that advanced age is positively associated with the severity of infection and sepsis [42], [43]. The probable reason for this was that lymphocyte function decrease with increasing age may increase the risk of infection and overwhelming secretion of type 2 cytokines may produce an overstimulation of viral replication, eventually leading to body function impairment [44]–[46]. All in all, age is an important predictor of rehabilitation in patients with COVID-19.

White blood cells (WBCs) are blood cells that mediate the immune system and resist exotic microorganisms. There are five types of WBCs, including neutrophils, lymphocytes, monocytes, eosinophils, and basophils [47]. Neutrophilic granulocyte is the chief phagocytic white blood cell of the blood, comprising 50-70% of the total leukocytes in the blood [48]. Neutrophilic granulocytes play a momentous role in the unspecific immune response and inflammation [49], [50]. Neutrophilic granulocytes are the first line to defense microbial pathogens, especially in pyogenic bacteria. Lymphocytes originate from the spleen, tonsils and lymph nodes are the smallest of the white blood cells, comprising 20%–40% of the total leukocytes in blood. Lymphocytes are a necessary component of the immune response of human body, which is involved in the synthesis and distribution of antibodies in the blood [51]. The B-lymphocytes play a core role in humoral immunity or antibody immunity, and the T-lymphocytes are responsible for cellular immunity. A number of clinical observations have suggested that a reduced number of lymphocytes and an increased number of neutrophils commonly occur in COVID-19 patients [1], [52], [53]. As an example, a population-based study of more than 100 patients suggested that severe COVID-19 patients had higher neutrophil count and lower lymphocyte count during the severe phase [54]. Liu and colleagues also found that lymphopenia and neutrophil count increased in patients with COVID-19 upon hospital admission may indicate more severe acute lung injury. Therefore, lymphopenia, neutrophil count increases may be a strong predictor of disease severity [55]. There is reason to believe that severe COVID-19 patients have immune disorders and excessive inflammation. Thus, patients with COVID-19 were rapidly screened with excessive inflammation using routine laboratory parameters in order to enhance survival rates. Hence, in recent years, N/L has been a simple parameter to reflect easily patient’s inflammatory [56]. Meanwhile, N/L was widely used as a simple and inexpensive marker for monitoring the infectious diseases, including patients with acute-on-chronic hepatitis B, pulmonary tuberculosis and bacterial community-acquired pneumonia [57], [58]. Furthermore, N/L also has been considered to be a strong independent prognostic indicator for a variety of diseases including malignant tumors, cardiovascular diseases, sepsis, chronic obstructive pulmonary disease, and infectious disease [59]–[62]. Elevated N/L was associated with poor clinical prognosis [63]–[65]. Elevated N/L has been used as a marker in the determination of increased inflammation in acute exacerbations of chronic obstructive pulmonary disease, which is similar to C-reactive protein [66]. N/L seemed to be a safe, simple and promising marker in predicting bacteremia and in grading community-acquired pneumonia, and N/L was more accurate and objective than C-reactive protein [67]. A large population-based retrospective cohort study from London reported that more than 3,500 people living with human immunodeficiency virus cases proved elevated N/L was an independent predictor of the risk of cardiovascular disease rather than a risk traditional risk factor for cardiovascular disease [68]. In line with these findings, there are several studies describing the relationship between N/L and COVID-19 patients. Lagunas-Rangel *et al.* revealed that N/L was significantly higher in the severe COVID-19 patients compared with the non-severe group, and the elevation of N/L is closely associated with poor prognosis in COVID-19 patients [69]. Liu and colleagues also demonstrated that the incidence of severe patients aged 50 years or older and N/L ≥ 3.13 was up to 50%. Similar to the findings of their study, we also found that N/L was significantly higher in severe COVID-19 patients displaying about 2.9 times higher levels than in non-severe COVID-19 patients. Taken together, N/L was shown to be of significant predictive value for the discrimination of COVID-19 patients’ status and might predict COVID-19 progression.

Platelets are viewed as important immune cells in the human body. Platelets have important physiological functions in the human body. Platelets have multiple physiological functions in the human body, including hemostasis, coagulation, the maintenance of vascular integrity, angiogenesis, innate immunity, inflammatory responses, viral infection, and malignant tumor. The number of platelets and platelet activity is strongly associated with multiple diseases [70]. Platelets are the smallest cell in peripheral blood cells, generating from megakaryocytes in the bone marrow [71] In recent years, research has shown that platelets and lymphocytes play a key role in the inflammatory response process. P/L as a novel type of inflammation factor has attracted more and more attention from researchers. P/L could serve as an indicator of inflammation that may reflect the level of systemic inflammation in patients [72]. P/L serves a role in a wide spectrum of diseases, including acute ischemic stroke, urothelial carcinoma, acute pulmonary embolism and Pulmonary large cell neuroendocrine carcinoma [73]–[76]. Thus, P/L might be a reliable prognostic marker in the progression and prognosis of many diseases. Wang et al carried out a retrospective study that included 134 adenosquamous cell lung cancer and found that high P/L was closely related to shorter disease-free survival and lower overall survival rates [77]. Wang and colleagues revealed that elevated P/L was an independent predictor for poor prognosis of patients with hepatocellular carcinoma [78]. Wang *et al.* also reported that of the 695 lung cancer cases, lower P/L was significantly associated with a lower tumor-node-metastasis (TNM) stage and a low incidence rate of surgery [79]. Based on the results of previous research, there is a reason to believe the relationship between the P/L and COVID-19. Qu and colleagues revealed that high P/L was positively correlated with COVID-19 severity and hospital lengths of stay. They believed that P/L may provide a useful indicator for monitoring the patients with COVID-19. In this study, we found that P/L in severe COVID-19 patients was about 2.0 times higher compared to non-severe COVID-19 patients. Thus, P/L has the potential to be used as a predictive marker for COVID-19 disease severity. Until now, there are very few reports describing the cost-effective markers N/L and P/L to joint predict the severity and prognosis of the infectious disease. To the best of our knowledge, this is the first time that the combined of age, N/L and P/L for predicting the prognosis of patients with COVID-19 using a machine learning method.

However, there are some limitations in this study. First of all, our data set was based on a single-center and the sample size was not large enough. In order to improve diagnostic accuracy, we will enlarge the sample size in future research. Second, multi-center researches will be necessary to carry out external validation for the RF-SMA-SVM model. Third, we plan to include more blood indexes in our future research.

## V. Conclusion and Future Works

In this study, a validated RF-SMA-SVM model was developed to differentiate the severity of COVID-19 based on factors such as basic patient information and 26 blood routine indicators. The main innovations of this paper are: on the one hand, it is proposed for the first time that the severity of COVID-19 can be distinguished by using the combination of age, N/L and P/L indicators, and on the other hand, it is the first time that the SMA is used to train an optimal SVM classifier. Based on the experimental results, the proposed method exhibits higher prediction accuracy and more stable performance on the COVID-19 severity prediction problem than the SVM method based on other optimization algorithms, while screening out the key factors rich in discriminatory power.

In future work, we will attempt to apply the proposed RF-SMA-SVM model to the COVID-19 pre-diagnosis problem and then attempt to apply it to other disease prediction problems.
